# Does the Mode of Plastid Inheritance Influence Plastid Genome Architecture?

**DOI:** 10.1371/journal.pone.0046260

**Published:** 2012-09-27

**Authors:** Kate Crosby, David Roy Smith

**Affiliations:** 1 Department of Biology, Dalhousie University, Halifax, Nova Scotia, Canada; 2 Canadian Institute for Advanced Research, Department of Botany, University of British Columbia, Vancouver, British Columbia, Canada; University Of Montana - Missoula, United States of America

## Abstract

Plastid genomes show an impressive array of sizes and compactnesses, but the forces responsible for this variation are unknown. It has been argued that species with small effective genetic population sizes are less efficient at purging excess DNA from their genomes than those with large effective population sizes. If true, one may expect the primary mode of plastid inheritance to influence plastid DNA (ptDNA) architecture. All else being equal, biparentally inherited ptDNAs should have a two-fold greater effective population size than those that are uniparentally inherited, and thus should also be more compact. Here, we explore the relationship between plastid inheritance pattern and ptDNA architecture, and consider the role of phylogeny in shaping our observations. Contrary to our expectations, we found no significant difference in plastid genome size or compactness between ptDNAs that are biparentally inherited relative to those that are uniparentally inherited. However, we also found that there was significant phylogenetic signal for the trait of mode of plastid inheritance. We also found that paternally inherited ptDNAs are significantly smaller (n = 19, *p* = 0.000001) than those that are maternally, uniparentally (when isogamous), or biparentally inherited. Potential explanations for this observation are discussed.

## Introduction

Plastids originate from an ancient endosymbiosis of a cyanobacterium by a eukaryotic host [1]. They first arose in the ancestor of the Archaeplastida (i.e., Plantae), and were then passed on laterally to diverse lineages through eukaryote-eukaryote endosymbioses [2], [3]. The genomes within contemporary plastids show a remarkable, and puzzling, diversity of sizes (5 to >1000 kilobases; kb) and compactnesses (<5 to >85% noncoding DNA) [4]. However, the evolutionary forces that gave rise to this variation are poorly understood.

The mutational hazard hypothesis argues that large, bloated genomes, with lots of intergenic and intronic DNA, pose a greater mutational burden to their hosts than genomes that are compact [5]. This is because any expansion in DNA content increases the potential for deleterious mutations, where the higher the mutation rate the greater the burden of having excess DNA. It follows, therefore, that species with large effective genetic population sizes (*N_e_*), where natural selection is efficient, are better at perceiving and eliminating “burdensome” excess DNA than those with a small *N_e_* [5]. Many studies have explored the relationship between *N_e_* and genome compactness [6]–[8], but few have employed plastid DNA (ptDNA).

Effective genetic population size is a difficult parameter to measure, and one that is likely influenced by the mode of inheritance. Plastid genomes, unlike most nuclear chromosomes, are typically uniparentally inherited [9]. For sexually reproducing species with male and female gametes, maternal plastid inheritance is the norm. Studies, however, have identified diverse species with paternal or biparental modes of plastid inheritance [10]–[13]. Other things being equal, the *N_e_* of uniparentally inherited plastid genomes should be half that of biparentally inherited ones. Further, the influence of differential migration (e.g. seeds are heavier and less numerous than pollen) and an individual’s size at reproduction (e.g. smaller individuals produce greater amounts of pollen vs. seeds) mean that maternal vs. paternal modes of organellar inheritance can also lead to overall differences in the *N_e_* of ptDNAs [14].

In this study, we use newly available data on plastid genome sequence and inheritance pattern to investigate how differing modes of inheritance impact ptDNA architecture. Based on the mutational hazard hypothesis, we predict that biparentally inherited ptDNAs, given their potential for having a higher *N_e_*_,_ will be more compact than those that are uniparentally inherited. We also expect to see differences in genomic architecture between paternally vs. maternally vs. uniparentally (when isogamous) inherited ptDNAs.

## Methods

By searching the literature, we found 81 species for which both plastid inheritance statistics and complete ptDNA sequence data are available, including 69 land plants, 6 green algae, 2 red algae, 2 apicomplexans, and 2 stramenopile (Table 1). The mode of plastid inheritance is thought to vary continuously rather than discretely between taxa; however, determining an appropriate scale for ranking the degree of biparental inheritance was difficult because of large differences in sample sizes between species. Instead, we categorized the primary pattern of plastid inheritance using the following: inheritance determined from genetic analysis of mutant plastids; ptDNA restriction analysis and/or analysis of ptDNA sequence data of progeny with known parentage; epifluorescence microscopy employing DNA fluorochromes to detect plastids in viable, mature sperm cells; and ultrastructural observations using transmission electron microscopy (TEM). Further, we noted cases where interspecific, intergeneric, or widely divergent strain cross was used to assess plastid mode of inheritance because at least one previous study has shown that taxonomically divergent crosses can cause the breakdown of the typical pattern of cytoplasmic maternal inheritance [15]. In a few cases the primary mode of inheritance was undetermined for the species with a complete plastid sequence in our dataset, so we screened the literature for plastid inheritance studies from other members of the same genera or higher-level taxonomic group; if the mode of inheritance was identical within the group, then we assumed all members from that group had the same mode of plastid inheritance (e.g. maternal inheritance for the genus *Cuscuta* or paternal inheritance for the order *Pinales*).

**Table 1:** Organisms, coarse taxonomic group, plastid genome size, coding proportion of ptDNA, primary mode of plastid inheritance, and references to support the mode of inheritance.

Organism	Accession #	TaxonomicGroup	Plastid GenomeSize (bp)	Codingproportion	PrimaryInheritance	Reference
Cicer arietinum	NC_011163	Land Plant	125319	0.52	Biparental	[10], [37] *
Ectocarpus siliculosus	NC_013498	Stramenopile	139954	0.74	Biparental	[38], [39] *
Equisetum arvense	NC_014699	Land Plant	133309	0.54	Biparental	[40]
Geranium palmatum	NC_014573	Land Plant	155794	0.37	Biparental	[10]
Ipomoea purpurea	NC_009808	Land Plant	162046	0.54	Biparental	[41], [42]
Medicago truncatula	NC_003119	Land Plant	124033	0.53	Biparental	[43]
Oenothera argillicola	NC_010358	Land Plant	165055	0.49	Biparental	[10], [44]
Oenothera biennis	NC_010361	Land Plant	164807	0.49	Biparental	[10], [44]
Oenothera elata subsp. hookeri	NC_002693	Land Plant	165728	0.49	Biparental	[10], [44]
Oenothera glazioviana	NC_010360	Land Plant	165225	0.49	Biparental	[10], [44]
Oenothera parviflora	NC_010362	Land Plant	163365	0.49	Biparental	[10], [44]
Pelargonium×hortorum	NC_008454	Land Plant	217942	0.52	Biparental	[10], [45]
Phaseolus vulgaris	NC_009259	Land Plant	150285	0.54	Biparental	[10], [46] *
Pisum sativum	NC_014057	Land Plant	122169	0.53	Biparental	[10], [47]–[49]
Psilotum nudum	NC_003386	Land Plant	138829	0.65	Biparental	[50]
Selaginella moellendorffii	NC_013086	Land plant	143780	0.54	Biparental	[51]
Trifolium subterraneum	NC_011828	Land Plant	144763	0.39	Biparental	[52]
Solanum lycopersicum	NC_007898	Land Plant	155461	0.58	Maternal	[53]
Arabidopsis thaliana	NC_000932	Land Plant	154478	0.51	Maternal	[10], [54]
Bryopsis hypnoides	NC_013359	Green Algae	153429	0.35	Maternal	[55]
Carica papaya	NC_010323	Land Plant	160100	0.49	Maternal	[56] *
Chara vulgaris	NC_008097	Green Algae	184933	0.48	Maternal	[57]
Cheilanthes lindheimeri	NC_014592	Land Plant	155770	0.52	Maternal	[58]
Coffea arabica	NC_008535	Land Plant	155189	0.51	Maternal	[10]
Cucumis sativus	NC_007144	Land Plant	155293	0.5	Maternal	[10], [59] *
Cuscuta exaltata	NC_009963	Land Plant	125373	0.48	Maternal	[10]
Cuscuta gronovii	NC_009765	Land Plant	86744	0.61	Maternal	[10]
Cuscuta obtusiflora	NC_009949	Land Plant	85286	0.6	Maternal	[10]
Cuscuta reflexa	NC_009766	Land Plant	121521	0.49	Maternal	[10]
Cycas taitungensis	NC_009618	Land Plant	163403	0.55	Maternal	[60] *
Daucus carota	NC_008325	Land Plant	155911	0.5	Maternal	[61], [62]
Eimeria tenella	NC_004823	Apicomplexan	34750	0.67	Maternal	[63]
Ephedra equisetina	NC_011954	Land Plant	109518	0.66	Maternal	[33]
Eucalyptus globulus subsp. globulus	NC_008115	Land Plant	160286	0.5	Maternal	[64] *
Fragaria vesca subsp. vesca	NC_015206	Land Plant	155691	0.53	Maternal	[65]
Fucus vesiculosus	NC_016735	Stramenopile	124986	0.79	Maternal	[66]
Ginkgo biloba	NC_016986	Land Plant	156988	0.42	Maternal	[67]
Glycine max	NC_007942	Land Plant	152218	0.51	Maternal	[10]
Gossypium hirsutum	NC_007944	Land Plant	160301	0.49	Maternal	[68]
Gracilaria tenuistipitata	NC_006137	Red Algae	183883	0.82	Maternal	[69]
Helianthus annuus	NC_007977	Land Plant	151104	0.51	Maternal	[70]
Hordeum vulgare subsp. vulgare	NC_008590	Land Plant	136462	0.44	Maternal	[71], [72]
Lolium perenne	NC_009950	Land Plant	135282	0.44	Maternal	[73]
Manihot esculenta	NC_010433	Land Plant	161453	0.45	Maternal	[10]
Nicotiana tabacum	NC_001879	Land Plant	155943	0.54	Maternal	[74]
Olea europaea	NC_013707	Land Plant	155888	0.53	Maternal	[75]
Oryza sativa Indica Group	NC_008155	Land Plant	134496	0.36	Maternal	[10]
Oryza sativa Japonica Group	NC_001320	Land Plant	134525	0.49	Maternal	[10]
Panicum virgatum	NC_015990	Land Plant	139619	0.43	Maternal	[76] *
Populus trichocarpa	NC_009143	Land Plant	157033	0.53	Maternal	[77] *
Porphyra purpurea	NC_000925	Red Algae	191028	0.81	Maternal	[78] *
Ricinus communis	NC_016736	Land Plant	163161	0.49	Maternal	[10]
Silene vulgaris	NC_016727	Land Plant	151583	0.53	Maternal	[79]
Solanum tuberosum	NC_008096	Land Plant	155296	0.53	Maternal	[10]
Sorghum bicolor	NC_008602	Land Plant	140754	0.42	Maternal	[10]
Toxoplasma gondii	NC_001799	Apicomplexan	34996	0.6	Maternal	[80]
Triticum aestivum	NC_002762	Land Plant	134545	0.45	Maternal	[10]
Vitis vinifera	NC_007957	Land Plant	160928	0.49	Maternal	[10]
Volvox carteri	GU084820	Green Algae	461064	0.2	Maternal	[81] *
Zea mays	NC_001666	Land Plant	140384	0.48	Maternal	[82] *
Cathaya argyrophylla	NC_014589	Land Plant	107122	0.57	Paternal	[33], [83]–[87] *
Cedrus deodara	NC_014575	Land Plant	119299	0.53	Paternal	[33], [83]–[87] *
Cephalotaxus wilsoniana	NC_016063	Land Plant	136196	0.58	Paternal	[33], [83]–[87] *
Cryptomeria japonica	NC_010548	Land Plant	131810	0.56	Paternal	[88]
Keteleeria davidiana	NC_011930	Land Plant	117720	0.54	Paternal	[33], [83]–[87] *
Larix decidua	NC_016058	Land Plant	122474	0.5	Paternal	[89]
Picea morrisonicola	NC_016069	Land Plant	124168	0.48	Paternal	[90] *
Picea sitchensis	NC_011152	Land Plant	120176	0.37	Paternal	[90] *
Pinus contorta	NC_011153	Land Plant	120438	0.49	Paternal	[33], [83]–[87] *
Pinus gerardiana	NC_011154	Land Plant	117618	0.51	Paternal	[33], [83]–[87] *
Pinus koraiensis	NC_004677	Land Plant	117190	0.54	Paternal	[33], [83]–[87] *
Pinus krempfii	NC_011155	Land Plant	116989	0.51	Paternal	[33], [83]–[87] *
Pinus lambertiana	NC_011156	Land Plant	117239	0.52	Paternal	[33], [83]–[87] *
Pinus monophylla	NC_011158	Land Plant	116479	0.52	Paternal	[33], [83]–[87] *
Pinus nelsonii	NC_011159	Land Plant	116834	0.52	Paternal	[33], [83]–[87] *
Pinus thunbergii	NC_001631	Land Plant	119707	0.62	Paternal	[33], [83]–[87] *
Pseudotsuga sinensis var. wilsoniana	NC_016064	Land Plant	122513	0.56	Paternal	[33], [83]–[87] *
Taiwania cryptomerioides	NC_016065	Land Plant	132588	0.62	Paternal	[33], [83]–[87] *
Chlamydomonas reinhardtii	NC_005353	Green Algae	203828	0.39	Uniparental	[91] *
Nephroselmis olivacea	NC_000927	Green Algae	200799	0.63	Uniparental	[92]
Zygnema circumcarinatum	NC_008117	Green Algae	165372	0.51	Uniparental	[93]

* Evidence for plastid inheritance in one or more studies listed was obtained from an interspecific or widely divergent strain cross.

Noncoding ptDNA content was calculated as follows: genome length minus the collective length of all annotated protein-, rRNA-, and tRNA-coding regions, not including the portions of these regions that are also annotated as introns. Intronic and non-standard open reading frames were treated as noncoding DNA. This method is contingent on the authors of the GenBank records having properly annotated their entry.

We performed a linear regression between plastid genome length (independent variable) and the amount of noncoding ptDNA (dependent variable). Both variables were log-transformed to meet the assumptions of homoscedasticity and normality. To test the effect of plastid inheritance pattern on noncoding ptDNA content and plastid genome size, we performed two non-parametric analyses. The factor “plastid inheritance” contained four levels: biparental vs. maternal vs. paternal vs. uniparental isogamous. The first analysis tested how all four levels affected the dependent variables (using separate Kruskal-Wallis tests for each variable). For the second analysis, we pooled the last three levels into ‘uniparental’ and used Wilcoxon rank sign tests. We applied non-parametric tests because our data were not normally distributed and because of the uneven sample sizes between levels of the factor “mode of plastid inheritance.” When more than two levels were used, we looked for significant differences between the various levels by performing *post-hoc* multiple comparisons using the Kruskal-Wallis test (function ‘kruskalmc’ in the R package ‘pgirmess’). Statistical analyses were performed with R v.2.14.2 (R Core Development Team 2012).

### Phylogenetic Independent Contrasts and Phylogenetic Signal in Our Dataset

Because our dataset was comprised of several groups of very closely related species (Table 1), we considered if the effects of phylogenetic non-independence (and by proxy pseudoreplication) [16], [17] were influencing the conclusions from our initial analyses. First we checked the tree topology of our dataset using a taxonomic tree generated from the NCBI Taxonomy Database [18], [19], and a maximum-likelihood phylogeny (10000 bootstraps using the PhyML plugin for Geneious Pro v. 5.4.4 [20]) based on the deduced amino acid sequences of the plastid-encoded *rbcL* gene (see Table 1 for GenBank accession numbers). Both trees had identical topologies except that the *rbcL* tree contained no apicomplexans because their ptDNAs do not contain *rbcL*. Because most tests of phylogenetic independence require a tree to be rooted, we forcibly rooted our *rbcL* tree in the red algal species *Gracilaria tenuistipitata var. liui*.

Phylogenetic independent contrasts (PICs) for the continuous variables of ptDNA size and noncoding content were performed using the ‘crunch’ function within the ‘caper’ package [20] of R v.2.14.2 (R Core Development Team 2012). To investigate the association between plastid genome size and noncoding ptDNA content, we fit a linear model of the standardized contrasts against each other. We were unable to obtain a large number of contrasts for our dataset that incorporated all nodes of the phylogeny (taxonomic or gene tree) for the categorical variable of primary mode of inheritance. This is because the tips of our phylogeny did not possess sufficient variation in the categorical trait, and with categorical variables only the tips are used in assessing the role of phylogenetic non-independence [21], [22]. Instead, we performed an analysis of phylogenetic signal strength (*D*) [23] for the binary trait of biparental vs. uniparental plastid inheritance to see if these traits were “clumped” or randomly distributed [22], [23] in the phylogeny. *D* values that are negative or close to 0 are more phylogenetically conserved (or clumped), which can indicate non-independent evolutionary events, whereas *D* values closer to 1 are overdispersed and therefore can be a sign of randomness in the trait’s distribution within a phylogeny.

## Results and Discussion

### As Plastid Genome Size Increases so does the Amount of Noncoding ptDNA

**Figure 1:**
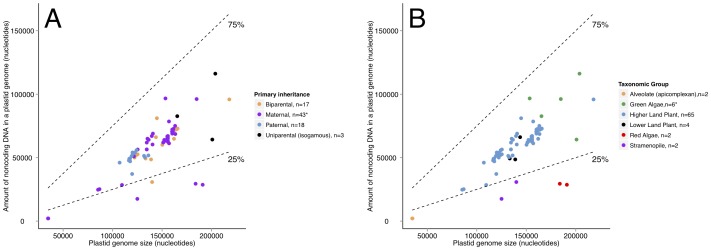
Amount noncoding ptDNA regressed on plastid genome size with mode of inheritance indicated and amount noncoding ptDNA regressed on plastid genome size with major taxonomic group indicated. Dashed lines on both figures indicate the 25% and 75% bounds for percent of noncoding DNA in a plastid genome. Analysis was carried out with all taxa (n = 82), and with logged variables. *We present the raw data here with *Volvox carteri* not pictured for ease of visual display (n = 81).

Consistent with previous observations [5], [24], the amount of noncoding ptDNA in nucleotides co-varied positively with plastid genome size for our dataset (*n* = 81), adjusted R^2^ = 0.78, p≤0.000001 (Fig. 1 A and B). Logged transformation of both variables enabled our linear model to meet the more crucial assumption for linear regression – homoscedasticity, but transformation did not improve normality. There was one significant high-leverage outlier (*Volvox carteri*) and two moderate statistical outliers (the apicomplexans *Toxoplasma gondii* and *Eimeria tenella*). Removal of these statistical outliers from our dataset (*n* = 78) did not alter the significance of the linear relationship, adjusted R^2^ = 0.76, p≤0.000001. When we fit a linear model to our standardized phylogenetic independent contrasts there was still a positive significant relationship (p = 0.00078) between plastid genome size and amount noncoding ptDNA, but the strength of the relationship decreased, adjusted R^2^ = 0.136. The assumptions of homoscedasticity and normality were violated in fitting this linear model, and neither log transformation of the variables nor the removal of the high-leverage outlier *Volvox carteri* helped us meet these assumptions. Overall, we contend that if more taxa were added to our dataset, this pattern would remain consistent with the past observations that plastid genome size scales positively with the amount of noncoding ptDNA [24].

### Plastid Genome Size and Compactness do not Vary Significantly between Taxa with Biparental vs. Uniparental Plastid Inheritance Patterns

her plastid genome size nor the amount of noncoding ptDNA varied significantly with respect to the primary mode of plastid inheritance when only two types of inheritance pattern were considered (uniparental vs. biparental) (plastid genome size: Wilcoxon signed rank test 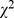 = 2, df = 1, p-value = 0.12; noncoding ptDNA: Wilcoxon signed rank test 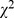 = 2, df = 1, p-value = 0.23). Our analysis of phylogenetic signal strength revealed that the binary trait of mode of plastid inheritance was clumped, *D* = −0.0052, and the probability that this trait was distributed at random in the phylogeny is effectively zero. This is likely due to the pseudoreplication produced from including multiple species of the same genus (e.g. *Oenothera, Pinus, Cuscuta, Picea*). Reducing our dataset, by randomly including only one taxon from each of the pseudoreplicated genera produced no significant difference between biparental and uniparental taxa (Wilcoxon signed rank test, df = 1, p-value range = 0.32–0.54). We expected uniparentally-inherited plastids, because of their potential for a reduced *N_e_*, to have more bloated ptDNAs than those with biparentally inherited ones, especially when looking within lineages. Our results suggest that forces other than, or in addition to, inheritance pattern are influencing *N_e_*_(ptDNA)_ and ultimately shaping plastid genome architecture.

Population bottlenecks can severely reduce the effective population size of a species [25]. Our dataset includes many crop and model species (e.g., *Triticum aestivum* and *Arabidopsis thaliana*), including some that show biparental plastid inheritance (e.g., *Pisum sativum* and *Medicago truncatula*). In the process of being bred for “desirable traits” or under laboratory conditions, it is likely that these species experienced multiple and frequent bottlenecks, which may have greatly reduced *N_e_*_(ptDNA)_ and canceled out the slight increases in *N_e_*_(ptDNA)_ due to biparental modes of plastid inheritance. Similarly, several of the taxa showing biparental plastid inheritance are the products of hybridizations – events that can alter genome architecture and size [26]. Indeed, the hybrid *Pelargonium*×*hortorum* (the garden geranium) has a very large ptDNA genome (217 kb), and one that is thought to have been shaped by one or many hybridization events [27]. In contrast, *Geranium palmatum*, a close relative of *Pelargonium*×*hortorum* but not a hybrid, has a relatively small ptDNA genome (156 kb).

It has also been argued that biparental organelle inheritance as compared to uniparental inheritance is more likely to cause the rapid spread of deleterious cytoplasmic elements (such as a mutant organelle genome with a replication advantage over the wild-type genome) through a sexual population [28]. Although our study was not designed test this particular hypothesis, our observation that ptDNA architecture did not vary significantly with respect to the primary mode of plastid inheritance does not support the view that biparental organelle inheritance promotes the spread of selfish cytoplasmic elements.

### Reduced ptDNA Size for Species with Paternally Inherited Plastomes: Lineage Specific Gene Loss or Male-biased Mutation?

**Figure 2:**
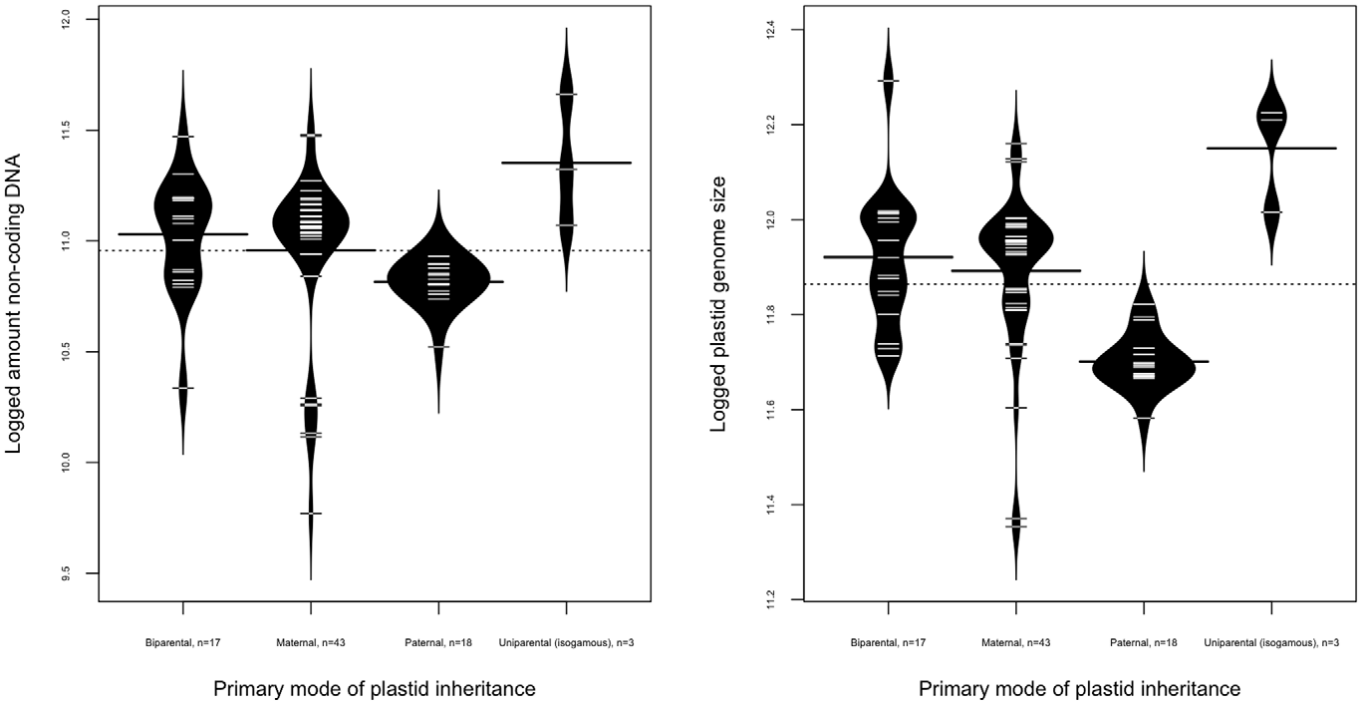
Beanplot in left panel depicts the difference in the amount of logged noncoding DNA content between four modes of plastid inheritance. Beanplot in right panel depicts the difference in the logged total plastome size between the four modes of plastid inheritance. The dashed line in the middle of each of the plots is the overall average of the continuous variable on the y-axis. The thick black line in the middle of each level for the factor of primary inheritance is the median for the continuous variable. The black curved beanpod surrounding the observations “beans” is the theoretical probability density distribution of these observations (n = 78, outliers removed in figure, not analysis).

Both plastid genome size and compactness differed significantly with respect to plastid inheritance pattern when four different modes of inheritance were considered: biparental, uniparental isogamous, maternal, and paternal (Fig. 2) (plastid genome size: Kruskal-Wallis 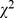 = 30.3, df = 3, p-value = 0.0000012; noncoding ptDNA: Kruskal-Wallis 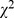 = 19.2, df = 3, p-value = 0.00025). *Post-hoc* tests revealed that paternally inherited plastid genomes are significantly smaller (plastid genome size) and more compact (amount of noncoding ptDNA) than plastid genomes inherited biparentally, maternally or through uniparental isogamous (critical probability level for post-hoc tests set at p = 0.001).

### Are Paternally Inherited ptDNAs Truly Smaller than those Following Other Patterns of Inheritance?

In our dataset, all of the taxa with paternally inherited plastid genomes belong to pinophytes (i.e., conifers). The ptDNAs of pinophytes tend to have fewer NADH dehydrogenase-encoding *ndh* genes (because of gene loss or gene transfer to the nuclear genome) than those from most other land plant lineages [29], [30], which largely explains their smaller sizes. Gnetophytes, which are close relatives of pinophytes, also have small plastid genomes with a reduced number of *ndh* genes [31]. However, unlike pinophytes, gnetophytes are believed to have maternally inherited plastids (at least for some *Ephedra* species) [32], [33], supporting the notion that the small ptDNAs within these two groups are probably the product of gene loss and not plastid inheritance pattern.

That said, male-biased mutation pressure [34]–[36] may also help to explain why pinophytes have smaller plastid genomes. It is well-established that male-biased mutation occurs in the biparentally inherited nuclear genomes of various animal taxa because male germ-lines cells go through many rounds of cell division, which means they are subjected to increased mutation rates compared to female germ-line cells. Female germ-line cells do not typically undergo cell division throughout the lifespan, and so are effectively buffered from the potentially deleterious effects of mutation. However, plants (unlike animals) were long hypothesized not to have a separation between germ-line and somatic cells, yet both nuclear- and plastid-encoded genes that are transferred paternally still undergo greater amounts of mutation compared to those that are maternally transmitted [34]–[36]. It is possible that paternally inherited plastid genomes have higher mutation rates because of male-biased mutation, and thus are potentially subject to more intense selection pressure for genome compaction [5].

### Concluding Remarks

Considering all of the data available at present, we have shown that the ptDNA genomic traits of size and compactness do not vary significantly with respect to mode of plastid inheritance, i.e. biparental vs. uniparental modes of inheritance. These observations are not in line with our expectations formulated under the mutational hazard hypothesis. We expected species with uniparentally inherited plastids to be larger and more bloated than biparentally inherited ones – they were not. However, we did find that paternally inherited ptDNAs were more compact and smaller than maternally and biparentally inherited plastid genomes. One hypothesis for this observation is that paternally inherited ptDNAs have a higher mutation rate due to male-biased mutation pressure. If true, this may mean that there is a greater “burden” associated with carrying excess DNA in plastid genomes that are paternally inherited relative to those that are maternally or biparentally inherited.
